# Emotions and decisions in the real world: What can we learn from quasi-field experiments?

**DOI:** 10.1371/journal.pone.0243044

**Published:** 2020-12-16

**Authors:** Syon P. Bhanot, Daphne Chang, Julia Lee Cunningham, Matthew Ranson

**Affiliations:** 1 Department of Economics, Swarthmore College, Swarthmore, PA, United States of America; 2 Heinz College of Information Systems and Public Policy, Carnegie Mellon University, Pittsburgh, PA, United States of America; 3 Management and Organization Area, Ross School of Business, University of Michigan, Ann Arbor, MI, United States of America; 4 Athenahealth, Watertown, MA, United States of America; Baylor University, UNITED STATES

## Abstract

Researchers in the social sciences have increasingly studied how emotions influence decision-making. We argue that research on emotions arising naturally in real-world environments is critical for the generalizability of insights in this domain, and therefore to the development of this field. Given this, we argue for the increased use of the “quasi-field experiment” methodology, in which participants make decisions or complete tasks after as-if-random real-world events determine their emotional state. We begin by providing the first critical review of this emerging literature, which shows that real-world events provide emotional shocks that are at least as strong as what can ethically be induced under laboratory conditions. However, we also find that most previous quasi-field experiment studies use statistical techniques that may result in biased estimates. We propose a more statistically-robust approach, and illustrate it using an experiment on negative emotion and risk-taking, in which sports fans completed risk-elicitation tasks immediately after watching a series of NFL games. Overall, we argue that when appropriate statistical methods are used, the quasi-field experiment methodology represents a powerful approach for studying the impact of emotion on decision-making.

## Introduction

Emotions are central to the human experience. Given this, understanding how emotions induced in real-world settings affect decision-making is of considerable importance. For example, how do anxiety and elation affect risk-taking by executives or investors? How do emotional events, such as marriage, divorce, or the death of a family member, influence how we behave in the days that follow? And how do natural disasters or pandemic events, and the negative emotions they induce, impact individuals coping with the devastation?

A large body of existing research has established that emotions affect cognitive processes related to risk perception and valuation, and that decision-makers are influenced by emotions unrelated to the decisions themselves [1–14]. A great deal of this work comes from laboratory research. In a typical study, lab participants are randomly induced to experience a particular mood or emotion before completing a decision task [15]. For example, researchers have manipulated emotions and moods through autobiographical recall tasks [16–18], newspaper stories [19], film clips [20], and pictures [21]. While such approach offers insight into the mechanisms of emotionally-fueled decisions, it is unclear whether the conclusions from laboratory settings, where emotions are induced rather than naturally triggered, generalize to the day-to-day contexts of people feeling emotions and their effects in the real world [22].

A second strain of literature seeks to address some of these concerns using retrospective quasi-experimental methods, which explore how real-world emotional shocks affect behavior captured through existing data [23–25]. For example, Card and Dahl [26] find that domestic violence reports increase in cities after unexpected home-team football game losses. While studies using this approach can be compelling, the quasi-experimental methodology is limited by real-world circumstances. In particular, researchers can only study how emotions and moods affect the types of choices that happened to occur after the event, and even then, only if data exists documenting the outcomes of those choices. Furthermore, researchers using this approach have to make assumptions about the emotional states that individuals experienced, since they are not actively engaging with or surveying individuals in most cases.

In this paper, we argue for greater use of a third approach: a “quasi-field experiment” methodology, in which researchers collect participants’ data using surveys and/or decision tasks in field settings, immediately after participants experience an emotion-inducing and quasi-random real-world event. Note that this can be thought of as a subset of the lab-in-the-field approach as defined by Gneezy and Imas [27]. Specifically, while we refer to field work using lab paradigms (which Gneezy and Imas [27] define as “lab-in-the-field”), we are interested in the subset of these studies that use a quasi-random real world event (rather than researcher-implemented randomization) to create (quasi-)experimental variation. These data collection efforts typically encompass both surveys of emotional state and standard laboratory protocols for measuring behavior and preferences (e.g., sets of gambles designed to measure risk aversion, or partner games designed to measure altruistic behavior). The key requirement is that the real-world event must contain an unpredictable component, such that participants’ emotions following the event are as good as randomly assigned. Importantly, studies of this type can involve collecting data from the same participant repeatedly over time, with the participant experiencing various emotional states. Examples of this approach in existing literature include studies that administer risk elicitation tasks to students following exams [28] to homeowners following natural disasters [29] and to individuals following exposure to violence [30].

Our paper makes two main contributions to the literature on emotions and decision-making. First, we conduct the first review of the emerging set of studies that use quasi-field experiments to study emotional carryover on decision-making. Our review shows that the quasi-field experiment methodology is at least as effective at inducing emotion as laboratory mood-induction procedures. However, our review also identifies a possible statistical weakness of some prior research in this area.

Second, based on this identified statistical shortcoming, we develop and demonstrate an improved statistical methodology, which we argue could inform better study designs for quasi-field experiments and inform how data from such studies are analyzed. In general, the quasi-field experiment research design is vulnerable to omitted variable bias. For example, even though an exam may influence students’ emotional state, students who perform poorly on a test may differ in unobservable ways from those who do well on a test—and those differences could influence their behavior in a subsequent decision task. In our review of the existing literature, we find that the majority of quasi-field experiment studies use statistical techniques that do not fully address this problem. In this paper, we present a solution, based on a formal statistical model. In particular, we show that a panel instrumental variables approach, with repeat data from each participant, can help address some of these statistical concerns. To the best of our knowledge, no previous study has used this approach or laid out a formal statistical model to provide a clear basis for causal inference in quasi-field experiments on emotion.

We demonstrate the methodology using a quasi-field experiment that explores the relationship between emotions induced by naturally-occurring events and risk preferences. Our study involves football fans completing risk-elicitation tasks immediately after a series of National Football League (NFL) playoff games. For our analysis, we utilize our proposed panel instrumental variable approach, and find evidence that negative emotions increase risk-taking behavior. Notably, we also find that individual characteristics are correlated with risk-taking behavior, which emphasizes the importance of using an appropriate statistical approach in quasi-field experiments.

We conclude that while there is great promise for quasi-field experiments as a tool to study emotions and decision making, it is critical for researchers to consider potential statistical issues with the approach and build them into their research methodologies and analysis. By doing so, we are confident that social scientists can develop a robust literature that shows us how naturally-occurring emotions impact real-world decisions.

### Review of methodological approaches

To provide context for our paper, this section reviews and compares the roles of laboratory and retrospective quasi-experimental methods in studying emotions and moods. We conclude this section with a comparison of these approaches to the more limited quasi-field experiments literature. Note that while there is no consensus on the definition of “emotions” (see Frijda [31] for a review), in the current paper, we draw from Schachter and Singer [32] and define emotions as “[states] of physiological arousal and of [cognitions] appropriate to [that] state of arousal.” In addition, we follow the distinction drawn by Clore and Ortony [33]; emotions are linked with personal evaluations of an event while moods are not. However, we defer to the authors’ use of terms when discussing their individual work in this section.

In addition, we limit our discussion of laboratory and field experiments to studies about the impact of emotions and moods on actual behavior. Although we limit our discussion in this way, we note that an alternative method of studying the impact of emotions and moods on behavior uses observational or survey data rather than behavioral data. For example, Krekel et al. [34] conducted a meta-analysis of research studies by Gallup to investigate an employee’s sense of well-being on productivity and performance. Daly et al. [35], on the other hand, utilized a combination of biological and psychological measurements and economics measures of time discounting to identify correlations between individual characteristics and time preferences.

Furthermore, we note that there have been several comprehensive meta-analyses that examine the generalizability of laboratory results (e.g. [36,37]). In addition, there have also been extensive discussions of the advantages and disadvantages of laboratory experiments and field experiments in economics [38–41].

#### Laboratory experiments

Laboratory experiments are often used to study the impact that emotions have on behavior. The selection criteria for this section are papers from either the economics or psychology literature that utilize emotion- or mood-induction methods that are frequently used in laboratory experiments. While we limit our review to commonly used methods in economics studies, there are other methods that have also been used to induce physiological and emotional responses in laboratory experiments (See Martin [42] for a review of other mood induction methods).

The most commonly used techniques to induce emotions or moods in this area of research include mood induction procedures (MIPs), autobiographical recalls, and success-failure experiences (SFEs). In MIPs, moods and emotions are induced using short readings, film clips, and pictures. For example, Conte et al. [43] used film clips to elicit specific emotions: joviality, sadness, anger, and fear. Similarly, Deldin and Levin [44] induced elated and depressed states by asking participants to read a series of 60 statements that were either progressively more positive or more negative. Autobiographical recall, meanwhile, leverages participants’ personal experiences as part of the procedure to induce specific emotions. For example, Fessler et al. [45] asked participants to recall or imagine situations where they experienced anger or disgust and then to write an essay about it. In contrast to MIPs and autobiographical recalls, SFEs induce emotions through manipulations of real-world outcomes rather than relying on recalls or participants’ imagination. The procedure asks participants to complete a task, then manipulate the outcome (or the perceived outcome) such that participants experience either success or failure from the task.

These methods allow researchers to manipulate moods and emotions in a controlled way to study their impact on preferences. For example, Stanton et al. [46] used film clips to induce happy, sad, or neutral mood to test the impact of mood and framing on risky choices. Ibanez et al. [14] used sets of images to induce amusement, awe, fear, and sadness, in order to test the impact of these emotions on generosity via a dictator game. Combinations of these methods are also used to further reinforce the success of the emotion or mood induction. For example, Andrade and Ariely [47] used a combination of film clips and autobiographical recall to study the extent to which positive and negative emotions influence behavior in ultimatum and dictator games. Similarly, Capra [48] utilized both autobiographical recall and SFE to induce good or bad moods, to study the impact of moods on choices in a dictator game, an ultimatum game, and a trust game.

Overall, the laboratory setting offers several important benefits relative to other techniques when studying emotions. First, laboratory studies provide greater control relative to field studies. Since laboratory participants are randomly assigned to different conditions in a controlled setting, researchers can more confidently assert that differences in participants’ subsequent decisions can be attributed to in-lab emotional manipulation. Second, there is clear evidence that the methods discussed in this section are successful in inducing specific emotions and moods. For example, there is evidence that some MIPs can induce similar levels of anxiety and depression to those experienced by clinically depressed participants [38]. And in a meta-analysis, Nummenmaa and Niemi [49] found that SFEs reliably induce both positive and negative emotions.

However, there are also disadvantages to conducting studies on emotion in laboratory settings. One important drawback to MIPs and the similar autobiographical recall method appears to be their susceptibility to demand effects. In a review of experimental papers on the effectiveness of mood induction procedures, Kenealy [50] found that these procedures and results are often inconsistent and subject to demand effects. Westermann et al.’s [51] meta-analysis of various MIPs also found significantly smaller mood induction effects in studies where subjects could guess or were told the purpose of the MIP. Similarly, while autobiographical recall certainly has greater ecological validity relative to the more traditional MIPs, the wording of instructions arguably encourages a demand effect (e.g. “Imagine that someone has done something to make you really angry. Briefly describe the circumstances that would make you the most angry,” from [45, p.114]). Because SFEs directly induce emotions by manipulating success or failure in the laboratory, this procedure is less susceptible to demand effects relative to both MIPs and autobiographical recalls. However, SFEs are limited in the types of emotions it can elicit (e.g. it would be difficult to induce fear in a laboratory setting using this method). In addition, note that the MIPs and SFE we discuss here were selected because they are frequently used in the literature. However, there exist other methods of inducing moods and emotions in the laboratory as well. For example, Cohn et al. [52] used the threat of an electric shock to induce fear in their participants, to test the impact that fear has on risk aversion.

Further, laboratory studies of emotion are generally limited in their external validity; that is, there is always the concern that insights gleaned from emotions induced in the lab (and the resulting behaviors they trigger) may not generalize to real-world contexts where emotions are experienced. For example, emotions are often experienced in the social context [53], which is limited in the laboratory setting. Therefore, a study testing the impact of a serious and personal emotional shock (say, from the death of a loved one) or a shared loss on a community may be difficult to replicate in a laboratory setting with existing MIPs or SFEs. Thus, while laboratory studies have strengths in this domain, it is important that it is supplemented by similar work from field settings.

#### Retrospective quasi-experiments

A second group of studies uses retrospective statistical techniques to measure how real-world behavior, captured by existing data, changes following emotionally charged real-world events. This methodology requires: (a) an event that causes an as-if random (therefore, “quasi-experimental”) emotional shock; (b) a relevant choice occasion that occurs following the event; and (c) data documenting the outcomes of those choices. S1 Table summarizes research that used random, natural-occurring events to retrospectively study the impact of emotions on subsequent behaviors in the field. The selection criteria we used was as follows: papers from either the economics or psychology literature that leverage real-world events as random shocks that induce emotions or moods, in order to retrospectively study the impact of emotions or moods on individual behavior following the shock.

Many studies in this literature utilize sports game outcomes as the real world shock, since fans tend to experience increased positive emotions after a home-team win and increased negative emotions after a home-team loss [54]. For example, Card and Dahl [26] used upset losses from home team professional football games as a negative emotional cue, in order to demonstrate a correlation between negative emotion and family violence. Similar work utilizes game outcomes to explore violence related to aggression [55,56], judicial sentencing [57], satisfaction with the political status quo [23,52], and risk aversion [25].

Another set of retrospective quasi-experimental studies rely on variation in weather conditions, based on evidence that weather significantly influences moods [58,59]. For example, Larrick et al. [59] found that high temperatures induce affective aggression, which is associated with greater hostile behavior by baseball pitchers (measured by pitchers hitting batters with pitches). Studies using weather as a natural, emotion/mood-inducing event have also found that weather affects payments at a Pay-What-You-Want restaurant [60], attribution bias [61], and projection bias [62]. Retrospective quasi-experimental studies also utilize other types of events, including terrorist attacks [63], sports outcomes [23,25,26,57], and deaths in the family [64–66].

Studies using the retrospective quasi-experimental approach can be compelling. Although this quasi-experimental approach lacks the control of laboratory studies, it utilizes real-world events and moods/emotions, which enhances generalizability. In addition, in contrast to the laboratory approach discussed earlier, this approach is not constrained by the type or intensity of moods and emotions induced by methods such as MIPs, SFEs, or autobiographical recalls.

However, researchers utilizing this quasi-experimental approach are limited by the datasets already in existence. As a result of this limitation, existing literature using retrospective quasi-experimental methods are constrained by the available measures in the dataset, which limits the types of mechanisms that researchers may study through which emotions might influence preferences and choices. Further, data on post-event emotional state is not always available, making it difficult to determine whether the event influenced decisions via emotions or some other pathway.

We note that there exists an alternative stream of literature that utilizes the quasi-experiment approach in which the main variable of interest is the direct impact of the random event on emotions and moods. There are also studies that leverage random events to examine their impact on behaviors that are reported through observations or surveys. In the current paper, we narrow our focus to approaches used to study the impact of emotions and moods on decision-making, but we include a sample of these other works in the S1 Appendix (S2 Table).

#### Quasi-field experiments

Quasi-field experiments, which combine the use of laboratory protocols with the use of real world events as quasi-random emotional shocks, combine some of the best features of laboratory studies and retrospective quasi-experiments. Like laboratory studies, quasi-field experiments use standard protocols to elicit preferences, values, and emotional states, e.g., via choice tasks and surveys. As a result, quasi-field experiments can be used to study many aspects of decision-making (risk aversion, altruism, cooperation, discounting, behavioral biases, etc.). However, unlike lab studies, quasi-field experiments utilize naturally occurring events that cause emotional shocks (like retrospective quasi-experiments). This allows researchers to study a greater variety of emotions and decisions relative to other methodologies, while retaining some external validity and control over process and measurement.

Table 1 summarizes quasi-field experiments that study the impact of emotions and moods on decision-making. The selection criteria we used was as follows: papers from either the economics or psychology literature that study the impact that emotions and moods have on preferences using real-world events as mood/emotion inducing shocks and laboratory elicitation instruments to measure preferences or behaviors ex-post. The table shows that this literature is nascent, with relatively few existing studies compared to the lab and retrospective approaches. We note that although we do not constrain the type of measured preferences in our review, the majority of the papers study risk preferences.

**Table 1:** Quasi-field experiments on emotions/moods and decision-making task.

Study	Research Question	Random event	Individual FE	IV regression
*Conflicts*				
Voors et al. [30]	The effect of exposure to conflict on social, risk, and time preferences.	Local conflicts	No	Yes
*Natural disasters*				
Cameron and Shah [67]	Effect of natural disasters on risk-taking behavior.	Natural disasters	No	No
Eckel et al. [68]	Effect of exposure to hurricane Katrina on risk preference over time.	Hurricane Katrina	No	No
Page et al. [29]	Effect of floods and losses in property values on risk aversion.	Floods/Natural disaster	No	Yes
*Exam feedback*				
Heilman et al. [28]	Effect of emotion regulation on decision making under risk.	Exam	No	No
*Weather*				
Bassi [69]	Effect of weather on the vote choice	Weather	No	No
*Misc*				
Guiso et al. [70]	Whether the 2008 financial crisis influenced investors' risk preference.	Financial crisis	No	No

The types of random events used to induce emotions in this literature are varied. For example, Heilman et al. [29] exploited exam performance as a shock to emotions, to explore how the induced emotions affected students’ risk preferences. Bassi [69] used variation in weather and a risk preference elicitation task to examine how weather-induced moods affected participants’ preference for voting for a political candidate whose performance is more uncertain. Voors et al. [30], meanwhile, tested how exposure to violence affects social preferences, risk preferences, and time preferences using laboratory instruments.

Other papers have utilized variation in natural disasters—including floods, earthquakes, and hurricanes—to examine how emotions influence risk-taking in decision tasks [30,67,68]. These papers employ familiar risk elicitation procedures to study changes in risk preferences in response to the emotion induced by the natural disaster. For example, Cameron and Shah [67] asked individuals to complete a task that involved selecting one gamble among a set of six gambles that varied in riskiness, after those individuals had been exposed to a flood or earthquake in Indonesia.

Quasi-field experiments offer a promising complement to laboratory and retrospective quasi-experimental studies. Importantly, our review of existing studies suggests that the magnitude of the emotional responses induced in field settings are comparable to those observed in the lab (see S3 Table). Furthermore, compared to the other, more widely-used methodologies in this space, quasi-field experiments have several strengths. The quasi-field experiment approach may be less susceptible to some of the methodological concerns discussed above with lab experiments. For example, using naturally-occurring events with quasi-field experiments might make it easier for researchers to mask their hypothesis to reduce the impact of demand effects [51]. Furthermore, the quasi-field experiment approach allows for the study of real world emotions, which might improve the generalizability of findings relative to controlled lab work. Additionally, in contrast to retrospective quasi-experiments, quasi-field experiments allow the researcher to study the effects of emotion on a wide variety of behaviors, as opposed to relying on the very limited set of decision contexts that occur and are documented following real-world events. Overall, quasi-field experiments are a valuable tool for researchers studying emotion and decision making, and we argue that the methodology merits more widespread use.

However, quasi-field experiments are by no means the perfect methodological tool. As with most quasi-experiments, quasi-field experiments provide less experimenter control relative to the laboratory approach. This lack of control means that there may be correlations between experienced emotions, decisions, and individual characteristics, which laboratory experiments largely circumvent through truly random assignment. To address these potential issues, researchers can include additional statistical tools, like repeat observations, instrumental variables approaches, and/or fixed-effects, to control for these potential confounds and omitted variables. However, our literature review finds that the majority of these studies have not incorporated either repeat observation, individual fixed-effects and/or IV regressions in their analysis (S1 and S2 Tables). Given that our approach uses instrumental variables, it is worth emphasizing that this approach depends on the use of a strong instrument [71]. If the instrument is only loosely correlated with the endogenous variable, it becomes difficult to defend a causal interpretation of its coefficient. A first stage F-test can be used to measure the strength of this correlation, which is equivalent to checking if the first-stage coefficients are equal to 0. In the next section, we discuss a statistical approach that incorporates these features, which we feel would help researchers develop quasi-field experiments that can convincingly uncover links between emotions and decisions.

### A statistical model for quasi-field experiments

In a typical laboratory experiment, researchers randomly assign participants to treatment and control groups. In contrast, quasi-field experiments rely on naturally-occurring emotional shocks. As a result, the statistical validity of their results depends heavily on whether it is possible to identify a source of emotional variation that is unrelated to participants’ characteristics. Our review of the literature suggests that many existing quasi-field experiment studies use techniques that are vulnerable to omitted variable bias to estimate the causal effect of emotions on decision making. To the best of our knowledge, studies either perform analyses with individual fixed effects or instrumental variables regression (or neither), but not both. The analyses in these studies, therefore, are susceptible to bias stemming from the fact that unobservable characteristics may be correlated with both the emotion-inducing event and the outcome measure. Note that the problem of omitted variable bias is by no means unique to this setting; many research designs have endogeneity challenges, which formal statistical approaches are designed to address. We put forward our model, and the research design it encourages, as an approach to address the specific endogeneity challenges inherent in field research on emotion.

For example, consider a risk preference study that relies on the emotions induced by exam performance. Even if exam performance strongly impacts emotional state, a comparison of risk-taking behavior after the exam would only be valid if students with good grades on the exam (or grades higher than they expected) were identical in all other respects to students with poor grades (or grades lower than they expected). Such an assumption is unlikely to be valid. In this paper, we propose a panel instrumental variable approach that can be used to help reduce these potential biases. We utilize the instrumental variable approach rather than a reduced form approach, because while the IV approach scales the reduced form, it also accounts for the statistical significance of the first stage regression. Unlike a reduced form regression, the instrumental variable regression requires both the first and second stage regressions to be statistically significant.

To demonstrate the issue and our proposed methodology to help solve it, a formal statistical model is useful. Consider the following model of how risk preferences depend on emotional state. Let *R*_*it*_ represent individual *i*’s risk-taking behavior at time *t*. Suppose that *R*_*it*_ is a function of current emotional state *E*_*it*_, an unobserved individual-specific personality characteristic *λ*_*i*_, an unobserved factor *Z*_*it*_, and an error term *ϵ*_*it*_:

$$R_{it}=\beta_{\text{1}}E_{it}+\beta_{\text{2}}Z_{it}+\lambda_{i}+\in_{it}$$ (1)

Suppose also that emotional state itself depends on the emotion-inducing event *M*_*it*_ (e.g. midterm exam score relative to expectations), an individual-specific personality characteristic *γ*_*i*_, the unobserved factor *Z*_*it*_, and an error term *ξ*_*it*_:

$$E_{it}=\alpha_{\text{1}}M_{it}+\alpha_{\text{2}}Z_{it}+\gamma_{i}+\xi_{it}$$ (2)

The goal of the study is to estimate *β*_1_, which represents the effect of emotions *E*_*it*_ on risk-taking behavior *R*_*it*_. The central challenge is that *E*_*it*_ is correlated with both omitted variables *γ*_*i*_ and *Z*_*it*_. Consider the midterm exam example: it is possible that both emotion and risk preferences could be correlated with personality characteristics such as stability or intelligence. Individual characteristics have been shown to correlate with how individuals experience and express emotions [72] For example, there is evidence that an individual’s gender has an impact on how that individual experiences an emotion [73,74]. Similarly, individual characteristics such as gender have also been shown to be correlated with risk-taking behavior [75,76]. Furthermore, it is also possible that some time-variant omitted variable (e.g., the time of day at which each student chooses to complete a follow-up risk-elicitation task) may influence both emotion and risk preferences. Thus, due to the omission of unobserved correlated variables, using cross-sectional or even panel data to estimate Eq (1) may result in a biased estimate of *β*_1_.

However, if—conditional on an individual-specific fixed effect—performance relative to expectations (*M*_*it*_) is orthogonal to the unobserved variable *Z*_*it*_, then *β*_1_ can be estimated using a panel instrumental variables approach that includes individual fixed-effects in both stages. This approach depends on the use of a strong instrument [71]. In other words, to avoid generating a biased measure of causal effect, the instrumental variable must strongly influence the endogenous variable (i.e., emotional state).

Although the panel instrumental variable approach is standard in some areas of economic research, few studies of emotions employ this method. Table 1 shows that few existing quasi-field experiment papers use either individual fixed effects or an instrument variable approach. To the best of our knowledge, no existing papers on this subject utilize a combination of both. Based on our model, we suggest that many quasi-field experiments and, more broadly, lab-in-the-field studies, that leverage emotional shocks as part of the design can be improved from a statistical perspective using a panel instrumental variable approach.

### An empirical example

To illustrate our statistical model, this section presents a study we conducted on emotions and risk preference. Specifically, we explore the effect of the emotional shocks induced by professional football (NFL) matches on sports fans’ risk preferences. While research using this quasi-field experiment approach has been conducted in the past, as we discussed earlier, our purpose in presenting this study is to show how a panel instrumental variable approach helps address some of the statistical challenges we identified in past work. Note that our study was reviewed and approved by the Harvard University IRB (F22473-102), and consent was obtained from all subjects.

Participants for this study were recruited through two online message boards for NFL football fans (www.thefantasyfootballguys.com and forums.footballguys.com). Participants were pre-screened through an online survey based on: (1) whether they completed the entire pre-screening survey; (2) how committed they were to their football team; (3) how available they were to complete post-game surveys; and (4) whether they had a PayPal account to receive payments. The initial email invitations were sent to 203 NFL fans, asking them to participate in a baseline survey for payment at their convenience, to be followed by further surveys immediately after upcoming NFL games. Participants had to complete the baseline survey in order to be part of the rest of the study, and 163 NFL fans ultimately participated in the study.

All participants were paid a guaranteed $5 show-up fee via PayPal after completing each survey, including the baseline survey. In addition, participants had the opportunity to win up to $26 in each survey, based on their choices in the risk elicitation tasks (the dollar values varied slightly depending on the payoffs and probabilities for each survey task).

The baseline survey sent to participants included more information about the study, solicited contact and basic demographic information from participants, and required participants to complete a risk-elicitation task modeled on the Balloon Analogue Risk Task (BART) from Lejuez et al. [77]. In order to measure the baseline level of risk-taking and to familiarize participants with the rules of the BART, all participants played three distinct “games” of the BART at baseline, for real money. The BART worked as follows. Each distinct BART game began with participants being presented with a virtual balloon, which had an initial dollar value to participants (either $1 or $2, as described below). During each of up to seven rounds of the game, the participant chose either to “inflate” or “not inflate” the balloon. If the participant inflated the balloon successfully, he or she could increase the dollar value of the balloon (by either $1 or $2). However, there was a small probability (known to the participant) that adding air to the balloon would make it pop. If the balloon did pop, the participant did not earn any money for that game. If the participant chose to stop inflating the balloon at any point before the balloon popped, the game ended and the participant kept the money he or she had earned up to that point.

We note that a limitation of this risk preference elicitation method is that we are not able to observe the choices that the participants would have made if the balloon had not popped, making ours an upper bound for how risk-averse (or a lower bound for how risk-loving) a person can be. We sought to minimize the impact of this issue by asking participants to play the BART game three times per participant per week and computing one risk aversion parameter for each participant for each week, with participants popping their balloon all three times in a given week only 18.1% of the time.

Following the baseline survey, each study participant was invited to complete up to six similar “post-game” surveys with BART, after NFL games in late 2012 and early 2013. The number of post-game surveys a given participant completed depended on how many games his/her favorite team played in the postseason. This period covered the NFL playoffs and Super Bowl, when multiple teams were no longer playing, so there were many weeks where there were fewer than 163 participants.

The day before each game, participants were sent an email message reminding them that they would receive a short survey to complete immediately after the football game. Then, the moment that their favorite team’s game ended, each participant received a text message and an email with a link to the post-game survey. The survey asked participants to self-report on six emotional states: excitement, nervousness, happiness, anger, sadness, and disappointment. It also asked them to complete three BART games, played for real money. Participants were given a 20-minute window after the game ended to complete the post-game survey. This ensured that their emotional response to the game was still strong at the time of survey completion. Table 2 summarizes the payoffs and probability of popping in the BART games, in each week of the survey. The payoffs and probability of popping varied slightly over the course of the study.

**Table 2:** Risk-elicitation task details for NFL fans study.

BART Game	Baseline	Week 1	Week 2	Week 3	Week 4	Week 5	Week 6
	***Initial Balloon Value***						
Games 1, 2, & 3	$1	$1	$2	$2	$2	$2	$2
	***Probability of Popping*, *Conditional on Choosing to Inflate***						
Game 1	0.25	0.25	0.25	0.25	0.25	0.25	0.25
Games 2 & 3	0.25	0.25	0.5	0.5	0.5	0.5	0.5
	***Increase in Value*, *Conditional on Choosing to Inflate and Not Popping***						
Game 1	+$1	+$1	+$1	+$1	+$1	+$1	+$1
Games 2 & 3	+$1	+$1	+$2	+$2	+$2	+$2	+$2

To construct our measure of emotion, we reduce the six emotional variable measures into a single measure using principal component analysis (PCA), which allows us to identify the main component of common variation between the specific emotion variables. Principal component analysis is often used in emotions studies in psychology [78,79] to reduce high-dimensioned responses (distinct measures of multiple emotions). This empirical technique is less frequently used by economists in studying emotions (see [80–82] for a few examples from economics, however). Importantly, our proposed statistical approach to quasi-field experiments does not rely on the use of PCA; rather, in this specific study, it was useful to employ PCA because the results show that the component vector with the largest eigenvalue has a natural interpretation as “negative emotion” (see S4 Table in the Supporting Information). To construct our final outcome variable, we project each participant’s emotional state onto this vector. Specifically, the negative emotion variable created using PCA includes approximately equal amounts of the anger, sadness, and disappointment variables, and an equal (but also negative) amount of the excitement and happiness variables.

To measure risk preferences, we use each decision by respondents in the Balloon Analogue Risk Task (BART) [77] to calculate a “risk aversion” parameter that measures the local curvature of the participant’s utility function. This parameter is based on the difference between the expected value of the gamble and the respondent’s certainty equivalent. We normalize by dividing this difference by the square root of the participant’s wealth in the losing state of the world. This normalization is intended to capture and adjust for the well-documented phenomenon that risk aversion tends to increase as stake sizes increase (see for example Holt and Laury [83] and Weber and Chapman [84], which document the relationship between stake size and risk aversion). Our normalization approach is a simple structural effort to recognize this pattern, but we acknowledge it is a coarse technique for building this intuitive pattern into our analysis. The parameter is defined as follows:

$$RiskAversion=\frac{(p_{w}W_{w}+(1-p_{w})W_{L})-W_{C}}{W_{L}^{0.5}}$$

In this equation, *p*_*w*_ is the probability of the winning outcome, *W*_*w*_ is the participant’s total wealth if the winning outcome occurs, *W*_*L*_ is her total wealth if the losing outcome occurs, and *W*_*c*_ is her wealth if she chooses the certain outcome instead of taking the gamble.

We use each participant’s set of choices in each survey (spanning 3 BART games) to calculate the value of the risk aversion parameter at which the respondent switches from the risky option (inflating the balloon) to the safe option (deciding not to inflate). This parameter is 0 for risk-neutral choices, greater than 0 for risk-averse choices, and less than 0 for risk-seeking choices. We then use that parameter value as the dependent variable (measure of risk-taking) in our subsequent analysis.

Note that in 80% of cases (at the participant-week level), we have at least one instance of the participant reaching a risky decision where they chose the safe option. In the remaining 20% of cases, participants always chose the risky option in all 3 BART games they played in that week, which makes our parameter estimate for them for that week an upper bound estimate of their risk aversion.

It is important to reiterate that these methodological approaches provide us with single-variable measures for both emotion and risk preference for each participant after each game they watched. Critically, the fact that we have multiple observations per individual allows us to utilize the panel instrumental variable approach.

**Fig 1:**
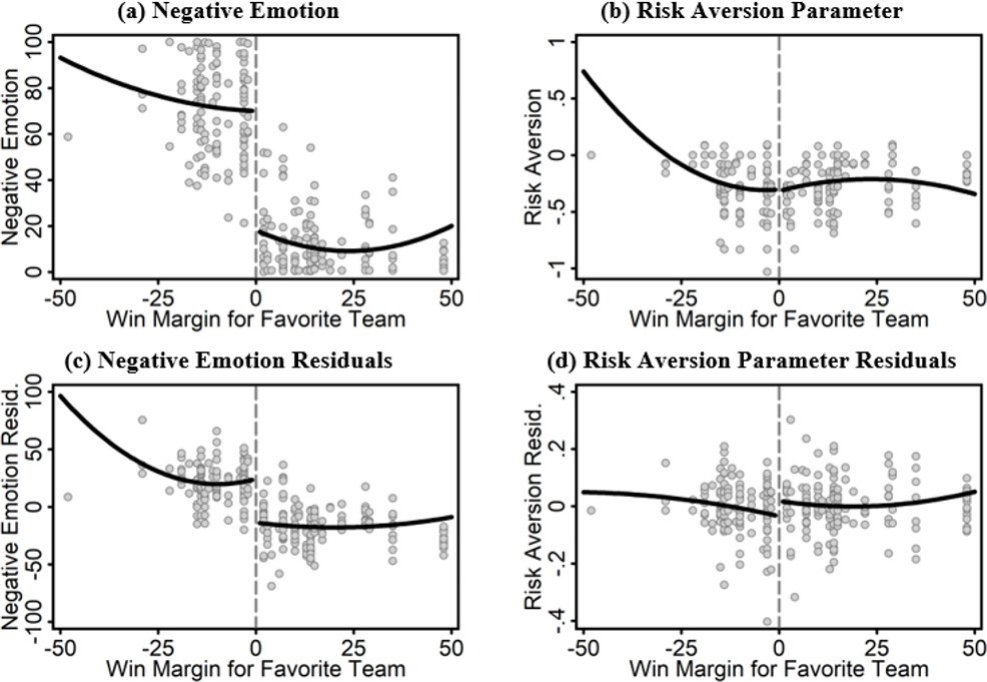
Outcomes by favorite NFL team win-loss margin. Notes: Each point in each panel represents a unique combination of participant and NFL game. The x-axis in each panel represents the win margin (in points) for the team that each fan cited as their favorite team. Negative values indicate losses. Panel (a) plots current negative emotion (immediately after the NFL game) as a function of win margin. Panel (b) plots risk aversion (measured using experimental tasks immediate after the NFL game) as a function of win margin. Panels (c) and (d) show residuals from regressions in which the same two outcome variables have been regressed on participant and survey fixed effects. The heavy solid line in each panel represents a regression of the outcome on win margin and win margin squared, allowing for separate intercepts and coefficients on either side of 0.

**Results from the NFL fans study. Evaluation of NFL game outcomes as an instrumental variable for emotion.** We begin by assessing the extent to which NFL game outcomes serve as an appropriate instrument for emotion (Fig 1). Two requirements must be satisfied. First, game outcomes must have strong, statistically significant effects on negative emotion. Second, game outcomes must not be correlated with unobservable respondent characteristics that also influence risk preferences.

For our statistical approach to be valid in this case, two requirements must be met: 1) NFL games must induce strong emotional responses; and 2) NFL games only influence risk preferences “through” variation in emotional states (and not directly). Panels (a) and (c) of Fig 1 present evidence on the first requirement, through visual evidence on how game outcomes influence fans’ emotions. The impact is striking: when a fan’s favored team won a game, he or she reported substantially fewer negative emotions. This pattern is strong, regardless of the number of points by which the team won the game. To test this requirement formally, we regress negative emotion on whether the team won and by how much. Specifications 1A and 1B in Table 3 report these regressions. Even after controlling for individual fixed effects, the effect of winning on negative emotions is negative and highly significant.

**Table 3:** The effect of negative emotion on risk-taking behavior in the NFL fans study.

	Dependent Variable: Negative Emotion		Dependent Variable: Risk Aversion			
Variable	(1A)	(1B)	(2)	(3A)	(3B)	(4)
	IV Stage 1	IV Stage 1	OLS	IV Stage 2	IV Stage 2	Direct RD
Negative Emotion			-0.0003	-0.0003	-0.0014*	
			(0.0003)	(0.0002)	(0.0007)	
Team Won	-62.74**	-54.44**				0.0746
	(3.31)	(8.37)				(0.0620)
Win Margin		-0.3258			-0.0043	-0.0039
		(0.8967)			(0.0041)	(0.0059)
Win Margin Squared		-0.0015			0.0000	0.0000
		(0.0325)			(0.0001)	(0.0002)
Won*Win Margin		-0.2706			0.0014	0.0018
		(1.0493)			(0.0049)	(0.0067)
Won*Win Margin Sq.		0.0131			0.0001	0.0001
		(0.0350)			(0.0002)	(0.0002)
Observations	442	442	442	442	442	442
R-squared	0.934	0.936	0.905	-	-	0.908
Participant FE	Yes	Yes	Yes	Yes	Yes	Yes
NFL Game FE	Yes	Yes	Yes	Yes	Yes	Yes
IVs:						
*Team Won*	-	-	No	Yes	Yes	No
Close Game Weights	No	Yes	No	No	Yes	Yes

Notes: Columns (1A) and (1B) show the results of regressions in which negative emotion is the dependent variable. In Columns (2) through (4), risk aversion is the dependent variable. Each cell in the table shows a coefficient, with the corresponding standard error below in parentheses. All standard errors are clustered by participant. The close game weights place greater weight on football games that were decided by a small point margin.

* denotes p < .05;

** denotes p < .01.

The second requirement is harder to test. We offer one approach in S5 Table in Supplementary Information, which compares the characteristics of participants who are fans of winning versus losing teams. Our results suggest that while participants are generally similar along most observables, fans of losing teams seem to be younger and have lower incomes than fans of winning teams. This is a potential concern as it might suggest unobservable differences between the fans as well, and suggests that a panel instrumental variable approach using individual fixed effects is appropriate.

Of course, the key concern for our analysis is that unobservable participant characteristics could be related to both the emotion-inducing event (the game outcome) and risk preferences. The outcome of any particular game clearly involves some degree of chance. However, even in circumstances where the real-world emotion-inducing event is plausibly random, bias from selection or other causes might still exist in subtle ways. For example, fans of teams that often lose might root for such teams because they are drawn to underdogs or longshots, by disposition, a characteristic that may be both hard to measure and correlated with risk preferences. Therefore, when repeat observation is possible (as in this case), it should be used as part of a panel instrumental variables approach.

#### Emotions and risk preferences

We next explore how negative emotions influence risk aversion in the study, using several different approaches to illustrate our methodological point. Table 3 presents the results from our regressions. First, specification 2 shows a “naïve” regression in which risk aversion is regressed directly on negative emotion, without individual or time fixed effects. The coefficient on negative emotion is close to zero and not statistically significant. Next, specification 3A presents a “simple” IV regression approach. This specification uses win/loss status of the football game as an instrumental variable for negative emotion, but does not control for the football game win margin. Again, the effect of negative emotion on risk aversion is small and insignificant. These approaches would therefore suggest minimal evidence of a relationship between negative emotion and risk preference in this context.

Specification 3B presents our preferred panel instrumental variables approach. In this specification, we again use win/loss status of the football game as an instrumental variable for negative emotion. However, we also control for the margin by which the football team won the game, and use weights that are inversely proportional to that margin. This has the effect of placing greater emphasis on games that were “cliff-hangers,” in which the football team won or lost by only a few points. This specification suggests that negative emotion has a negative effect on risk aversion (p < .05). Further, the point estimate is larger than in the previous specifications.

Finally, as a robustness check, specification 4 shows the results of estimating a regression discontinuity design that uses win margin as the running variable. Panels (b) and (d) in Fig 1 help to visualize the intuition for this approach, by plotting the relationship between win margin and risk aversion. While there is a strong decrease in negative emotion at the point where score difference is zero, there is no statistically significant discontinuity in risk aversion at the threshold.

Overall, when using our preferred panel instrumental variable approach, our results suggest that negative emotion may indeed reduce risk aversion. Notably, our empirical approach shows the importance of the panel instrumental variable method, namely that it allows for a less-biased estimate of the relationship between emotion and risk preference. Indeed, our approach influenced the point estimate and statistical significance in the analysis relative to more common empirical approaches. While there remain limitations to our approach (as discussed further in the next section), we believe that our approach helps reduce bias in estimates and thereby helps researchers better recover true relationships between emotion and decisions.

## General discussion

In the section above, we utilize a study as an example to demonstrate how a panel instrumental variables approach can help address omitted variable concerns in quasi-field experiments. We find that there are individual characteristics that may be correlated with both risk preferences and emotional states. Without an appropriate statistical approach, the coefficients might have underestimated the effect of negative emotion on risk aversion. For this reason, we argue that future quasi-field experimental research on emotions and decision making would greatly benefit from using an instrumental variables approach with panel data when possible.

There are limitations to our approach in this specific study that are important to highlight here, however. First, the use of an instrumental variable approach requires several assumptions. For this sports fans study, the most tenuous of these assumptions is the exclusion restriction, namely that our approach assumes that the game outcomes did not impact risk preference through any channel other than emotion. In this case, it is plausible that this assumption does not hold; for example, the game outcomes might have had impacts on other important variables that could influence risk preferences (i.e., income via gambling). To some extent, using panel data about an individual can help alleviate this concern, by allowing for data collection to test for these alternate channels at multiple moments in time (i.e., by asking whether and how much participants had bet on the game in question in each week). In our study, we did not ask explicitly about betting on the game in question, so we are limited in our ability to directly address this issue. However, researchers deploying our suggested empirical approach in the future should think, ex-ante, about possible violations of the exclusion restriction and include questions about possible alternative channels, to restrict bias in their estimates as much as possible.

Second, the construction of an individual-level panel dataset necessarily requires eliciting repeated measures. As a result, the study is susceptible to the common issues associated with repeated, self-reported measures (e.g. changes in expectations/perceptions over time or the possibility that subjects will strive for choice consistency across the repeat measures). In our case, this concern is somewhat alleviated by the large amount of time between games (~one week). However, it is surely possible that this limitation prevents the recovery of a wholly unbiased estimate (for example, if participants feel compelled to make their risky choices consistent week-to-week, it might reduce variance in the outcome variable and thereby hide potential links between emotion and risk preferences).

Third, the likelihood that a participant completes a given survey may be influenced by the expected/actual performance of his/her team, which could also bias the results. For example, if participants do not respond to surveys when their teams suffer a loss when they were expected to win, we would lose data from high negative emotion observations. This could reduce variance in a key variable in the analysis, negative emotion, and therefore make it more difficult to recover the true relationship between emotion and decision making.

Fourth, it is challenging to directly interpret effect sizes in our results. That is, because the measures are sensitive to the specific experimental design used, and the specific ways that we measured negative emotion and risk preferences, it is more difficult to directly compare our empirical results to those in the broader literature. That said, we present these results not as definitive proof of links between emotion and risk preferences, but rather to support our broader methodological argument that quasi-field experimental research designs that accommodate panel instrumental variable approaches are valuable for studying emotions and decision making.

## Conclusion

Quasi-field experiments are a promising and underused methodology for research on emotions and decision-making. In this paper, we argue that these types of hybrid studies are at least as effective at inducing emotions as laboratory studies. They offer an approach for studying intense emotions that cannot ethically be induced in a laboratory setting, while also providing greater flexibility than “pure” quasi-experimental research. Combined with appropriate statistical models, quasi-field experiments can produce rigorous inference about the causal effect of emotions on decisions, greatly enhancing the literature on this important topic.

The business of everyday life is ripe with opportunities for quasi-field experimental research on judgment and decision making. Indeed, numerous events—from sporting events to the news to personal triumphs and tragedies—impact human emotions. We argue that by taking advantage of naturally-occurring events in the field and utilizing appropriate statistical techniques, future research could deepen our understanding of how emotions influence the economic choices that individuals make.

## Supporting information

S1 Table Retrospective quasi-experimental research on emotions/moods and real-world behavior [citations listed at end of supporting information]. (DOCX)

S2 Table Additional quasi-experimental research on emotions/moods and real-world behavior [citations listed at end of supporting information]. (DOCX)

S3 Table Effect sizes of moods and emotions in selected laboratory and quasi-experiment studies [citations listed at end of supporting information]. (DOCX)

S4 Table Principal components of emotion. (DOCX)

S5 Table Summary statistics for NFL fans study. (DOCX)

S1 Appendix NFL fans study surveys. (DOCX)

S1 File (CSV)

S2 File (DTA)

## References

[1] Rottenstreich Y, Hsee CK. Money, kisses, and electric shocks: On the affective psychology of risk. Psychol Sci. 2001 5;12(3):185–90. 10.1111/1467-9280.00334 11437299

[2] Lerner JS, Keltner D. Beyond valence: Toward a model of emotion-specific influences on judgement and choice. Cogn Emot. 2000 7 1;14(4):473–93.

[3] Tiedens LZ, Linton S. Judgment under emotional certainty and uncertainty: the effects of specific emotions on information processing. J Pers Soc Psychol. 2001 12;81(6):973 10.1037//0022-3514.81.6.973 11761319

[4] Leith KP, Baumeister RF. Why do bad moods increase self-defeating behavior? Emotion, risk tasking, and self-regulation. J Pers Soc Psychol. 1996 12;71(6):1250 10.1037//0022-3514.71.6.1250 8979390

[5] Isen AM, Patrick R. The effect of positive feelings on risk taking: When the chips are down. Organ Behav Hum Perform. 1983 4 1;31(2):194–202.

[6] Mittal V, Ross WT Jr. The impact of positive and negative affect and issue framing on issue interpretation and risk taking. Organ Behav Hum Decis Process. 1998 12 1;76(3):298–324. 10.1006/obhd.1998.2808 9878512

[7] Yuen KS, Lee TM. Could mood state affect risk-taking decisions? J Affect Disord. 2003 6 1;75(1):11–8. 10.1016/s0165-0327(02)00022-8 12781345

[8] Brief AP, Butcher AH, Roberson L. Cookies, disposition, and job attitudes: The effects of positive mood-inducing events and negative affectivity on job satisfaction in a field experiment. Organ Behav Hum Decis Process. 1995 4 1;62(1):55–62.

[9] Small DA, Lerner JS, Fischhoff B. Emotion priming and attributions for terrorism: Americans' reactions in a national field experiment. Polit Psychol. 2006 4;27(2):289–98.

[10] Loewenstein G. Emotions in economic theory and economic behavior. Am Econ Rev. 2000 5;90(2):426–32.

[11] Gneezy U, Imas A, Madarász K. Conscience accounting: Emotion dynamics and social behavior. Manage Sci. 2014 8 8;60(11):2645–58.

[12] Celse J, Galia F, Max S. Are (negative) emotions to blame for being positional? An experimental investigation of the impact of emotional states on status preferences. J Behav Exp Econ. 2017 4 1;67:122–30.

[13] Campos-Vazquez RM, Cuilty E. The role of emotions on risk aversion: a prospect theory experiment. J Behav Exp Econ. 2014 6 1;50:1–9.

[14] Ibanez L, Moureau N, Roussel S. How do incidental emotions impact pro-environmental behavior? Evidence from the dictator game. J Behav Exp Econ. 2017 2 1;66:150–5.

[15] Coan JA, Allen JJ, editors. Handbook of emotion elicitation and assessment. Oxford university press; 2007 4 19.

[16] Callen M, Isaqzadeh M, Long JD, Sprenger C. Violence and risk preference: Experimental evidence from Afghanistan. Am Econ Rev. 2014 1;104(1):123–48.

[17] Lerner JS, Keltner D. Fear, anger, and risk. J Pers Soc Psychol. 2001 7;81(1):146 10.1037//0022-3514.81.1.146 11474720

[18] Lerner JS, Small DA, Loewenstein G. Heart strings and purse strings: Carryover effects of emotions on economic decisions. Psychol Sci. 2004 5;15(5):337–41. 10.1111/j.0956-7976.2004.00679.x 15102144

[19] Johnson EJ, Tversky A. Affect, generalization, and the perception of risk. J Pers Soc Psychol. 1983 7;45(1):20.

[20] Gross JJ, Levenson RW. Emotion elicitation using films. Cogn Emot. 1995 1 1;9(1):87–108.

[21] Lang PJ. International affective picture system (IAPS): Affective ratings of pictures and instruction manual. Technical report. 2005.

[22] Harrison GW, List JA, Towe C. Naturally occurring preferences and exogenous laboratory experiments: A case study of risk aversion. Econometrica. 2007 3;75(2):433–58.

[23] Miller MK. For the win! The effect of professional sports records on mayoral elections. Soc Sci Q. 2013 3;94(1):59–78.

[24] Hirshleifer D, Shumway T. Good day sunshine: Stock returns and the weather. J Finance. 2003 6;58(3):1009–32.

[25] Berument MH, Ceylan NB. Effects of soccer on stock markets: The return–volatility relationship. Soc Sci J. 2012 9 1;49(3):368–74.

[26] Card D, Dahl GB. Family violence and football: The effect of unexpected emotional cues on violent behavior. Q J Econ. 2011 2 1;126(1):103–43. 10.1093/qje/qjr001 21853617 PMC3712874

[27] Gneezy U, Imas A. Lab in the field: Measuring preferences in the wild. In Handbook of economic field experiments. 2017 Jan 1 (Vol. 1, pp. 439–464). North-Holland.

[28] Heilman RM, Crişan LG, Houser D, Miclea M, Miu AC. Emotion regulation and decision making under risk and uncertainty. Emotion. 2010 4;10(2):257 10.1037/a0018489 20364902

[29] Page L, Savage DA, Torgler B. Variation in risk seeking behaviour following large losses: A natural experiment. Eur Econ Rev. 2014 10 1;71:121–31.

[30] Voors MJ, Nillesen EE, Verwimp P, Bulte EH, Lensink R, Van Soest DP. Violent conflict and behavior: a field experiment in Burundi. Am Econ Rev. 2012 4;102(2):941–64.

[31] Frijda N. H. Moods, emotion episodes, and emotions. Handbook of Emotions. 1993; 381–403.

[32] Schachter S, Singer J. Cognitive, social, and physiological determinants of emotional state. Psychol. 1962;69(5): 379. 10.1037/h0046234 14497895

[33] Clore GL, Ortony A. Cognition in emotion: Always, sometimes, or never. Cognitive neuroscience of emotion. 2000; 24–61.

[34] Krekel C, Ward F, De Neve JE. Employee wellbeing, productivity, and firm performance. Saïd Business School WP 4. 2019.

[35] Daly M, Harmon CP, Delaney L. Psychological and biological foundations of time preference. J Eur Econ Assoc. 2009; 7(2–3): 659–669.

[36] Anderson CA, Lindsay JJ, Bushman BJ. Research in the psychological laboratory: Truth or triviality? Curr Dir Psychol Sci. 1999 2;8(1):3–9.

[37] Mitchell G. Revisiting truth or triviality: The external validity of research in the psychological laboratory. Perspect Psychol Sci. 2012 3;7(2):109–17. 10.1177/1745691611432343 26168439

[38] Levitt SD, List JA. On the generalizability of lab behaviour to the field. Can J Econ. 2007 5;40(2):347–70.

[39] Camerer C. The promise and success of lab-field generalizability in experimental economics: A critical reply to Levitt and List. Available at SSRN 1977749. 2011 Dec 30.

[40] Bardsley N. Experimental economics and the artificiality of alteration. J Econ Methodol. 2005 6 1;12(2):239–51.

[41] Kessler J, Vesterlund L. The external validity of laboratory experiments: The misleading emphasis on quantitative effects. Oxford, UK: Oxford University Press; 2015 1 2.

[42] Martin M. On the induction of mood. Clin Psychol Rev. 1990 1 1;10(6):669–97.

[43] Conte A, Levati MV, Nardi C. Risk preferences and the role of emotions. Economica. 2018 4;85(338):305–28.

[44] Deldin PJ, Levin IP. The effect of mood induction in a risky decision-making task. Bull Psychon Soc. 1986 7 1;24(1):4–6.

[45] Fessler DM, Pillsworth EG, Flamson TJ. Angry men and disgusted women: An evolutionary approach to the influence of emotions on risk taking. Organ Behav Hum Decis Process. 2004 9 1;95(1):107–23.

[46] Stanton S J, Reeck C, Huettel SA, LaBar KS. Effects of induced moods on economic choices. Judgm Decis Mak. 2014; 9(2): 167–175.

[47] Andrade EB, Ariely D. (2009). The enduring impact of transient emotions on decision making. Organ Behav Hum Decis Processes. 2009; 109(1), 1–8.

[48] Capra MC. Mood-driven behavior in strategic interactions. Am Econ Rev. 2004; 94(2): 367–372.

[49] Nummenmaa L, Niemi P. Inducing affective states with success-failure manipulations: A meta-analysis. Emotion. 2004 6;4(2):207 10.1037/1528-3542.4.2.207 15222857

[50] Kenealy PM. The Velten mood induction procedure: A methodological review. Motiv Emot. 1986 12 1;10(4):315–35.

[51] Westermann R, Spies K, Stahl G, Hesse FW. Relative effectiveness and validity of mood induction procedures: A meta‐analysis. Eur J Soc Psychol. 1996 7;26(4):557–80.

[52] Cohn A, Engelmann J, Fehr E, Maréchal MA. Evidence for countercyclical risk aversion: An experiment with financial professionals. Am Econ Rev. 2015; 105(2): 860–85.

[53] Coan JA, Allen JJB, eds. Handbook of emotion elicitation and assessment. Oxford university press, 2007.

[54] Wann DL, Dolan TJ, MeGeorge KK, Allison JA. Relationships between spectator identification and spectators' perceptions of influence, spectators' emotions, and competition outcome. J Sport Exerc Psychol. 1994 12 1;16(4):347–64.

[55] Rees DI, Schnepel KT. College football games and crime. J Sports Econom. 2009 2;10(1):68–87.

[56] Kirby S, Francis B, O’Flaherty R. Can the FIFA world cup football (soccer) tournament be associated with an increase in domestic abuse? J Res Crime Delinq. 2014 5;51(3):259–76.

[57] Eren O, Mocan N. Emotional judges and unlucky juveniles. Am Econ J. 2018; 10(3): 171–205.

[58] Cunningham MR. Weather, mood, and helping behavior: Quasi experiments with the sunshine samaritan. J Pers Soc Psychol. 1979 11;37(11):1947.

[59] Larrick RP, Timmerman TA, Carton AM, Abrevaya J. Temper, temperature, and temptation: Heat-related retaliation in baseball. Psychol Sci. 2011 4;22(4):423–8. 10.1177/0956797611399292 21350182

[60] Riener G, Traxler C. Norms, moods, and free lunch: Longitudinal evidence on payments from a Pay-What-You-Want restaurant. J Socio Econ. 2012 8 1;41(4):476–83.

[61] Haggag K, Pope DG, Bryant-Lees KB, Bos MW. Attribution Bias in Consumer Choice. Rev Econ Stud.

[62] Busse MR, Pope DG, Pope JC, Silva-Risso J. The psychological effect of weather on car purchases. Q J Econ. 2015 1 23;130(1):371–414.

[63] Metcalfe R, Powdthavee N, Dolan P. Destruction and distress: using a quasi‐experiment to show the effects of the September 11 attacks on mental well‐being in the United Kingdom. Econ J (London). 2011 2 1;121(550):F81–103.

[64] Meier AN. Emotions, risk attitudes, and patience. No. 1041. SOEPpapers on Multidisciplinary Panel Data Research. 2019.

[65] Liberini F, Redoano M, Proto E. Happy voters. J Public Econ. 2017; 146: 41–57.

[66] van den Berg GJ, Lundborg P, Vikström J. The economics of grief. The Economic Journal. 2017; 127(604): 1794–1832.

[67] Cameron L, Shah M. Risk-taking behavior in the wake of natural disasters. J Hum Resour. 2015 3 31;50(2):484–515.

[68] Eckel CC, El-Gamal MA, Wilson RK. Risk loving after the storm: A Bayesian-Network study of Hurricane Katrina evacuees. J Econ Behav Organ. 2009 2 1;69(2):110–24.

[69] Bassi A. Weather, risk, and voting: An experimental analysis of the effect of weather on vote choice. J Exp Polit Sci. 2019; 6(1): 17–32.

[70] Guiso L, Sapienza P, Zingales L. Time varying risk aversion. J Financ Econ. 2018 6 1;128(3):403–21.

[71] Bound J., Jaeger D., & Baker R. (1995). Problems with Instrumental Variables Estimation When the Correlation Between the Instruments and the Endogeneous Explanatory Variable is Weak. Journal of the American Statistical Association, 90(430), 443–450. 10.2307/2291055

[72] Gross JJ, John OP. Facets of emotional expressivity: Three self-report factors and their correlates. Pers Individ Dif. 1995 10 1;19(4):555–68.

[73] Fischer AH, Rodriguez Mosquera PM, Van Vianen AE, Manstead AS. Gender and culture differences in emotion. Emotion. 2004 3;4(1):87 10.1037/1528-3542.4.1.87 15053728

[74] Brody LR, Hall JA. Gender, emotion, and socialization InHandbook of gender research in psychology 2010 (pp. 429–454). Springer, New York, NY.

[75] Byrnes JP, Miller DC, Schafer WD. Gender differences in risk taking: a meta-analysis. Psychol Bull. 1999 5;125(3):367.

[76] Charness G, Gneezy U. Strong evidence for gender differences in risk taking. J Econ Behav Organ. 2012 6 1;83(1):50–8.

[77] Lejuez CW, Read JP, Kahler CW, Richards JB, Ramsey SE, Stuart GL, et al Evaluation of a behavioral measure of risk taking: the Balloon Analogue Risk Task (BART). J Exp Psychol Appl. 2002 6;8(2):75 10.1037//1076-898x.8.2.75 12075692

[78] Pettersson E, Turkheimer E. Approach temperament, anger, and evaluation: Resolving a paradox. J Pers Soc Psychol. 2013 8;105(2):285 10.1037/a0033046 23773039

[79] Torgerson WS. Theory and methods of scaling.

[80] Reuben E, van Winden F. Fairness perceptions and prosocial emotions in the power to take. J Econ Psychol. 2010; 31(6): 908–922.

[81] Treffers T, Koellinger PD, Picot A. Do Affective States Influence Risk Preferences?. Schmalenbach Business Review, 2016; 17(3–4), 309–335.

[82] Tyszka T, Przybyszewski K. Cognitive and emotional factors affecting currency perception. J Econ Psychol, 2006; 27(4): 518–530.

[83] Holt CA, Laury SK. Risk aversion and incentive effects. Am Econ Rev. 2002; 92(5): 1644–1655.

[84] Weber BJ, Chapman GB. Playing for peanuts: Why is risk seeking more common for low-stakes gambles? Organ Behav Hum Decis Process. 2005; 97(1): 31–46.

