# Supplementary material for: Emotions and decisions in the real world: What can we learn from quasi-field experiments?
Source: PLoS One. 2020 Dec 16;15(12):e0243044. doi: 10.1371/journal.pone.0243044 (PMC7744061; doi:10.1371/journal.pone.0243044)
Supplement: S1 Table — (DOCX) [file pone.0243044.s001.docx]

**Table S1: Retrospective quasi-experimental research on emotions/moods and real-world behavior [Citations Listed at End of Supporting Information]**

| **Study** | **Research Question** | **Random event** | **Individual FE** | **IV regression** |
| --- | --- | --- | --- | --- |
| *Sports outcome* |  |  |  |  |
| Berument and Ceylan [25] | Effect of soccer team success on stock market returns and volatility. | Sports outcome | No | No |
| Card and Dahl [26] | How football game outcomes influence family violence. | Sports outcome | No | No |
| Healy et al. (Study 1) [85] | Influence of information irrelevant to government performance on voting. | Sports outcome | No | No |
| Miller [23] | Effect of sports performance on incumbent mayoral elections. | Sports outcome | No | No |
| Eren [58] | Effect of game outcomes on judicial rulings. | Sports outcome | Yes | No |
| *Weather* |  |  |  |  |
| Cao and Wei [86] | Whether stock market returns are related to temperature. | Weather | No | No |
| Hirshleifer and Shumway [24] | The relationship between sunshine and daily market index returns. | Sunshine | No | No |
| Riener and Traxler [61] | The distribution and evolution of payments in a Pay-What-You-Want restaurant | Weather | No | No |
| Saunders [87] | How NYC weather influences daily changes in stock indexes. | Weather | No | No |
| Kamstra [88] | Whether there is a seasonal affective disorder effect in stock returns. | Seasons | No | No |
| Meier et al. [89] | How emotions affect the decision to keep or to change the status quo. | Weather | No | No |
| Simonsohn [90] | Whether the weather affects enrollment decisions. | Weather | No | No |
| *Miscellaneous* |  |  |  |  |
| Metcalfe et al. [64] | Spillover effect of 9/11 on people's subjective well-being in the UK. | Terrorist attack | Yes | No |

23. Miller MK. For the win! The effect of professional sports records on mayoral elections. Soc Sci Q. 2013 Mar;94(1):59-78.

24. Hirshleifer D, Shumway T. Good day sunshine: Stock returns and the weather. J Finance. 2003 Jun;58(3):1009-32.

25. Berument MH, Ceylan NB. Effects of soccer on stock markets: The return–volatility relationship. Soc Sci J. 2012 Sep 1;49(3):368-74.

26. Card D, Dahl GB. Family violence and football: The effect of unexpected emotional cues on violent behavior. Q J Econ. 2011 Feb 1;126(1):103-43.

58. Cunningham MR. Weather, mood, and helping behavior: Quasi experiments with the sunshine samaritan. J Pers Soc Psychol. 1979 Nov;37(11):1947.

61. Haggag K, Pope DG, Bryant-Lees KB, Bos MW. Attribution Bias in Consumer Choice. Rev Econ Stud.

64. Meier AN. Emotions, risk attitudes, and patience. No. 1041. SOEPpapers on Multidisciplinary Panel Data Research. 2019.

85. Healy AJ, Malhotra N, Mo CH. Irrelevant events affect voters' evaluations of government performance. Proc Natl Acad Sci. 2010 Jul 20;107(29):12804-9.

86. Cao M, Wei J. Stock market returns: A note on temperature anomaly. J Bank Financ. 2005 Jun 1;29(6):1559-73.

87. Saunders EM. Stock prices and Wall Street weather. Am Econ Rev. 1993 Dec 1;83(5):1337-45.

88. Kamstra MJ, Kramer LA, Levi MD. Winter blues: A SAD stock market cycle. Am Econ Rev. 2003; 93(1): 324-343.

89. Meier AN, Schmid L, Stutzer A. Rain, emotions and voting for the status quo. Eur Econ Rev. 2019; 119: 434-451.

90. Simonsohn U. Weather to go to college. The Economic Journal. 2010; 120(543): 270-280.
