# Supplementary material for: Emotions and decisions in the real world: What can we learn from quasi-field experiments?
Source: PLoS One. 2020 Dec 16;15(12):e0243044. doi: 10.1371/journal.pone.0243044 (PMC7744061; doi:10.1371/journal.pone.0243044)
Supplement: S2 Table — (DOCX) [file pone.0243044.s002.docx]

**Table S2: Additional quasi-experimental research on emotions/moods and real-world behavior [Citations Listed at End of Supporting Information]**

| **Study** | **Research Question** | **Random event** | **Individual FE** | **IV regression** |
| --- | --- | --- | --- | --- |
| *Exam feedback* |  |  |  |  |
| Butler and Mathews [92] | How does anxiety affect perception of probability of examination failure? | Exam | No | No |
| Krupić and Corr [93] | Effect of individual differences in eliciting emotions about exams. | Exam | Yes | No |
| *Sports outcome* |  |  |  |  |
| Healy et al. (Study 2) [85] | Influence of information irrelevant to government performance on voting. | Sports outcome | No | No |
| Hirt et al. [94] | Effect of sport outcomes on fan self-esteem and beliefs about future performances of fans and teams. | Sports outcome | No | No |
| *Weather* |  |  |  |  |
| Forgas et al. [95] | Can good or bad mood induced by the weather influence people's ability to correctly remember everyday scenes? | Weather | No | No |
| Guéguen [96] | How pleasant weather improves moods represented by nonverbal expressions. | Weather | No | No |
| Guéguen [97] | The effect of sunshine on courtship solicitation. | Weather | No | No |
| Guéguen and Lamy [98] | Does sunshine increase the likelihood of people spontaneously helping strangers? | Weather | No | No |
| *Miscellaneous* |  |  |  |  |
| Schaubroeck and Lam [99] | The attitudinal and behavioral effects of being promoted or rejected for promotion. | Promotion | No | No |

85. Healy AJ, Malhotra N, Mo CH. Irrelevant events affect voters' evaluations of government performance. Proc Natl Acad Sci. 2010 Jul 20;107(29):12804-9.

92. Butler G, Mathews A. Anticipatory anxiety and risk perception. Cognit Ther Res. 1987 Oct 1;11(5):551-65.

93. Krupić D, Corr PJ. Individual differences in emotion elicitation in university examinations: A quasi-experimental study. Pers Individ Dif. 2014 Dec 1;71:176-80.

94. Hirt ER, Zillmann D, Erickson GA, Kennedy C. Costs and benefits of allegiance: Changes in fans' self-ascribed competencies after team victory versus defeat. J Pers Soc Psychol. 1992 Nov;63(5):724.

95. Forgas JP, Goldenberg L, Unkelbach C. Can bad weather improve your memory? An unobtrusive field study of natural mood effects on real-life memory. J Exp Soc Psychol. 2009 Jan 1;45(1):254-7.

96. Guéguen N. Weather and courtship behavior: A quasi-experiment with the flirty sunshine. Soc Influ. 2013 Oct 1;8(4):312-9.

97. Guéguen N. Weather and smiling contagion: A quasi experiment with the smiling sunshine. J Nonverbal Behav. 2013 Mar 1;37(1):51-5.

98. Guéguen N, Lamy L. Weather and helping: additional evidence of the effect of the sunshine Samaritan. J Soc Psychol. 2013 Mar 1;153(2):123-6.

99. Schaubroeck J, Lam SS. Comparing lots before and after: Promotion rejectees' invidious reactions to promotees. Organ Behav Hum Decis Process. 2004 May 1;94(1):33-47.
