# Supplementary material for: Emotions and decisions in the real world: What can we learn from quasi-field experiments?
Source: PLoS One. 2020 Dec 16;15(12):e0243044. doi: 10.1371/journal.pone.0243044 (PMC7744061; doi:10.1371/journal.pone.0243044)
Supplement: S3 Table — (DOCX) [file pone.0243044.s003.docx]

**Table S3: Effect sizes of moods and emotions in selected laboratory and quasi-experiment studies [Citations Listed at End of Supporting Information]**

| **Study** | **Study type** | **Emotion/Mood induction** | **Affect scale** | **Type of emotion/mood** | **Effect Size (d)** |
| --- | --- | --- | --- | --- | --- |
| Larsen and Ketelaar [100] | Experiment | SFM | Bipolar scale | Happy/Sad | -0.322 |
| McFarland and Ross [101] | Experiment | SFM | Bipolar scale | Positive/Negative | 1.33 |
| Whitley [102] | Experiment | SFM | Bipolar scale | Happy/Unhappy | 1.708 |
| Fessler et al. [45] | Experiment | Autobiographical/  Imagination | PANAS | Anger | 1.413 |
|  |  |  |  | Disgust | 1.262 |
| Jallais and Gilet [103] | Experiment | Autobiographical recall | BMIS | Happiness | 0.406 |
|  |  |  |  | Serenity | 0.413 |
|  |  |  |  | Sadness | 1.123 |
|  |  |  |  | Anger | 0.89 |
| Kugler et al. [104] | Experiment | Autobiographical/  Imagination | Abbreviated PANAS | Anger | 1.221 |
|  |  |  |  | Fear | 0.852 |
| Phillips et al. [105] | Experiment | Autobiographical/  Imagination | Bipolar scale | Positive | 1.127 |
| Salovey [106] | Experiment | Autobiographical  /Imagination | 7-point scale | Happy | -1.024 |
|  |  |  |  | Sad | 1.327 |
| Conte et al. [43] | Experiment | Film clips | PANAS-X | Joviality | 0.06 |
|  |  |  |  | Sadness | 1.145 |
|  |  |  |  | Fear | 1.232 |
|  |  |  |  | Anger | 1.423 |
| Lerner et al. [18] | Experiment | Film clips | 9-point Likert scale | Sadness | -2.661 |
|  |  |  |  | Disgust | 4.32 |
| Treffers et al. [107] | Experiment | Film clips | Abbreviated PANAS-X | Joy | 1.56 |
|  |  |  |  | Fear | 1.3 |
|  |  |  |  | Sadness | 1.23 |
| Butler and Mathews [92] | Quasi-experiment | Exam | STAI-S | Anxiety (State) | 1.263 |
| Krupić and Corr [93] | Quasi-experiment | Exam return | PANAS | Positive | 1.055 |
|  |  |  |  | Negative | 0.058 |
| Forgas et al. [95] | Quasi-experiment | Weather | Bipolar scale | Positive/Negative | 1.6 |
| Parrott and Sabini [108] | Quasi-experiment | Exam return/recall | Unipolar scale | Happy | 0.567 |
| (Experiment 1) |  |  |  | Bad | 0.684 |
|  |  |  |  | Sad | 0.480 |
|  |  |  |  | Emotional | 0.105 |
|  |  |  |  | Confused | 0.281 |
|  |  |  |  | Ashamed-Proud | 0.569 |
|  |  |  |  | Angry-Grateful | 0.547 |
|  |  |  |  | Lonely-Belonging | 0.324 |
|  |  |  |  | Worried-Optimistic | 0.062 |
| (Experiment 2) | Quasi-experiment | Weather | 0-10 scale | Great mood | 0.857 |
|  |  |  |  | Bad mood |  |

18. Lerner JS, Small DA, Loewenstein G. Heart strings and purse strings: Carryover effects of emotions on economic decisions. Psychol Sci. 2004 May;15(5):337-41.

43. Conte A, Levati MV, Nardi C. Risk preferences and the role of emotions. Economica. 2018 Apr;85(338):305-28.

45. Fessler DM, Pillsworth EG, Flamson TJ. Angry men and disgusted women: An evolutionary approach to the influence of emotions on risk taking. Organ Behav Hum Decis Process. 2004 Sep 1;95(1):107-23.

92. Butler G, Mathews A. Anticipatory anxiety and risk perception. Cognit Ther Res. 1987 Oct 1;11(5):551-65.

93. Krupić D, Corr PJ. Individual differences in emotion elicitation in university examinations: A quasi-experimental study. Pers Individ Dif. 2014 Dec 1;71:176-80.

95. Forgas JP, Goldenberg L, Unkelbach C. Can bad weather improve your memory? An unobtrusive field study of natural mood effects on real-life memory. J Exp Soc Psychol. 2009 Jan 1;45(1):254-7.

100. Larsen RJ, Ketelaar T. Extraversion, neuroticism and susceptibility to positive and negative mood induction procedures. Pers Individ Dif. 1989 Jan 1;10(12):1221-8.

101. McFarland C, Ross M. Impact of causal attributions on affective reactions to success and failure. J Pers Soc Psychol. 1982 Nov;43(5):937.

102. Whitley Jr BE. The relationship of informational attributions to affective response to success and failure. J Soc Psychol. 1986 Aug 1;126(4):453-7.

103. Jallais C, Gilet AL. Inducing changes in arousal and valence: Comparison of two mood induction procedures. Behav Res Methods. 2010 Feb 1;42(1):318-25.

104. Kugler T, Connolly T, Ordóñez LD. Emotion, decision, and risk: Betting on gambles versus betting on people. J Behav Decis Mak. 2012 Apr;25(2):123-34.

105. Phillips LH, Bull R, Adams E, Fraser L. Positive mood and executive function: Evidence from Stroop and fluency tasks. Emotion. 2002 Mar;2(1):12.

106. Salovey P. Mood-induced self-focused attention. J Pers Soc Psychol. 1992 Apr;62(4):699.

107. Treffers T, Koellinger P, Picot AO. In the Mood for Risk? A Random-Assignment Experiment Addressing the Effects of Moods on Risk Preferences. ERIM Report Series Reference No. ERS-2012-014-ORG. 2012 Nov 21.

108. Parrott WG, Sabini J. Mood and memory under natural conditions: Evidence for mood incongruent recall. J Pers Soc Psychol. 1990 Aug;59(2):321.
