# Supplementary material for: Emotions and decisions in the real world: What can we learn from quasi-field experiments?
Source: PLoS One. 2020 Dec 16;15(12):e0243044. doi: 10.1371/journal.pone.0243044 (PMC7744061; doi:10.1371/journal.pone.0243044)
Supplement: S4 Table — (DOCX) [file pone.0243044.s004.docx]

| **Table S4: Principal components of emotion** | |
| --- | --- |
|  | **NFL Fans Study** |
| First Eigenvector |  |
| *Excited* | -0.22 |
| *Nervous* | 0.02 |
| *Anxious* |  |
| *Happy* | -0.44 |
| *Angry* | 0.49 |
| *Sad* | 0.50 |
| *Disappointed* | 0.52 |
| Eigenvalue | 2.28 |
| Observations | 163 |
| *Notes: Eigenvectors 2 through 6 had eigenvalues of 1.63, 0.78, 0.50, 0.44, and 0.37, respectively. Blank values in this table indicate that the study did not elicit information about that particular emotion.* | |
