# Supplementary material for: Emotions and decisions in the real world: What can we learn from quasi-field experiments?
Source: PLoS One. 2020 Dec 16;15(12):e0243044. doi: 10.1371/journal.pone.0243044 (PMC7744061; doi:10.1371/journal.pone.0243044)
Supplement: S5 Table — (DOCX) [file pone.0243044.s005.docx]

| **Table S5: Summary statistics for NFL fans study** | | | |
| --- | --- | --- | --- |
|  | **All Games** | **Games with Favorite Team Loss** | **Games with Favorite Team Win** |
| **Participant Characteristics** |  |  |  |
| Female | 0.04 (0.19) | 0.02 (0.15) | 0.05 (0.21) |
| Age ≥ 30 | 0.62 (0.49) | 0.57 (0.50) | 0.66 (0.48) |
| Household Income ($000s) | 72.11 (26.78) | 69.40 (27.33) | 74.29 (26.22) |
| Some College | 0.23 (0.42) | 0.24 (0.43) | 0.21 (0.41) |
| College Degree | 0.48 (0.50) | 0.49 (0.50) | 0.48 (0.50) |
| Graduate Degree | 0.27 (0.45) | 0.25 (0.43) | 0.29 (0.46) |
| Fan Level (1-5) | 3.52 (0.70) | 3.56 (0.67) | 3.48 (0.72) |
| NFL Games Watched Last Season: |  |  |  |
| *Favorite Team, on TV* | 14.47 (2.96) | 14.48 (2.91) | 14.45 (3.00) |
| *Favorite Team, Live* | 1.80 (2.21) | 1.88 (2.42) | 1.73 (2.04) |
| *Any Team, Live* | 2.44 (2.53) | 2.58 (2.80) | 2.32 (2.29) |
| **Football Game Outcomes** |  |  |  |
| Predicted Win Margin | 3.49 (6.53) | -0.59 (5.23) | 6.76 (5.57) |
| Actual Win Margin | 4.73 (17.48) | -10.59 (7.16) | 16.99 (13.04) |
| Team Won | 0.56 (0.50) | 0.00 (0.00) | 1.00 (0.00) |
| Percent of Game Watched | 88.67 (20.90) | 89.23 (20.70) | 88.23 (21.11) |
| **Post-Game Emotional State** |  |  |  |
| Excited | 44.16 (40.27) | 9.47 (19.71) | 71.92 (29.54) |
| Happy | 48.13 (42.13) | 7.33 (16.00) | 80.76 (24.23) |
| Sad | 26.42 (35.22) | 54.16 (35.56) | 4.23 (10.70) |
| Nervous | 18.20 (25.86) | 16.36 (25.67) | 19.67 (26.01) |
| Angry | 25.78 (34.51) | 50.31 (36.60) | 6.17 (14.46) |
| Disappointed | 41.61 (42.07) | 83.20 (22.23) | 8.34 (17.15) |
| Negative Emotion | 38.36 (33.84) | 71.80 (18.67) | 11.61 (12.95) |
| **Post-Game Choices and Outcomes** |  |  |  |
| Attempted Pumps per Balloon Game | 5.06 (2.55) | 4.89 (2.48) | 5.20 (2.61) |
| Risk Aversion Parameter | -0.25 (0.21) | -0.26 (0.22) | -0.24 (0.19) |
| Winnings per NFL Game ($) | 5.25 (3.49) | 5.35 (3.95) | 5.17 (3.08) |
| *Note: The table presents summary statistics for unique participant-by-NFL game observations. Each cell presents the mean value of the selected variable, with a standard deviation in parentheses.* | | | |
