# Supplementary material for: Emotions and decisions in the real world: What can we learn from quasi-field experiments?
Source: PLoS One. 2020 Dec 16;15(12):e0243044. doi: 10.1371/journal.pone.0243044 (PMC7744061; doi:10.1371/journal.pone.0243044)
Supplement: S1 Appendix — (DOCX) [file pone.0243044.s006.docx]

**S1 Appendix: NFL Fans Study Surveys**

**S1.A. Screening Survey**

Q1 This study is being conducted by Harvard researchers who are interested in how people make decisions.  We are looking for football fans who are willing to fill out short online surveys after three upcoming NFL games.  Each survey will take about five minutes, and participants will be paid based on their responses.  The average participant who completes all three surveys will earn about $50 total.

If you are interested in being part of this study and if you are at least 18 years old, please answer the following questions as honestly as possible.  We will determine your eligibility and will send you an email letting you know whether you meet the criteria for this study.

If at any time you decide that you do not want to be considered for this study, please close this browser window.  If you have other questions, you can contact the researchers in charge of this study at ppolharvard@gmail.com.

Q2 Which NFL team do you root for?

(List NFL teams)

Q3 How do you feel about the (SELECTED TEAM)?

o I am an extremely committed fan.  (1)

o I am a committed fan.  (2)

o I like this team.  (3)

o I think this team is OK.  (4)

Q5 How often do you feel the following emotions when the (SELECTED TEAM) lose?

|  | Never (1) | Rarely (2) | Sometimes (3) | Often (4) | Almost always (5) |
| --- | --- | --- | --- | --- | --- |
| Sad (1) | o | o | o | o | o |
| Angry (2) | o | o | o | o | o |
| Disappointed (3) | o | o | o | o | o |
| Frustrated (4) | o | o | o | o | o |

Q7 How many (SELECTED TEAM) games did you watch last season on TV?

o Almost every game  (1)

o Most games  (2)

o Some games  (3)

o A few games  (4)

Q12 Approximately how many (SELECTED TEAM) games have you attended in person over the last two years (this can include home or away games)?

o None  (1)

o One game  (2)

o Two to four games  (3)

o Five to ten games  (4)

o Ten or more games  (5)

Q16 Approximately how many professional NFL football games have you attended in person over the last two years, in total?

o None  (1)

o One Game  (2)

o Two to four games  (3)

o Five to ten games  (4)

o Ten or more games  (5)

Q15 Please answer the following trivia questions without looking up the answers.  If you don't know an answer, please leave the question blank.

Who was the (SELECTED TEAM)' starting quarterback on Opening Day of this season?

Q18 Who was the (SELECTED TEAM)' ' head coach on Opening Day of this season?

Q17 Who was the (SELECTED TEAM)' leading rusher (in total rushing yards) last season?

Q16 How many games did the (SELECTED TEAM)'  win during the regular season last year?

(Select 1-17)

Q17 What division are the (SELECTED TEAM) in?

(List NFL Conferences)

Q14 We are looking for people who honestly think they will have time to participate in this study.  Here is what the study requires:

Participants will complete a short online survey immediately after upcoming three NFL games.  Each survey will take about five minutes, and must be completed within 20 minutes of the end of each game.  The survey can be completed on the computer or on a smartphone. Participants will be paid $5 for each completed survey, and will have the chance to win up to $26 additional per survey.

If you are selected, will you have time to fill out an online survey after three upcoming NFL games?

o Yes, I will be likely to fill out a short survey immediately after each game.  (1)

o Maybe (I'm not sure).  (2)

o No, I will be unlikely to fill out a short survey immediately after each game.  (3)

End of Block: Sports Questionnaire

Start of Block: Demographics

Q41 What is your gender?

o Male  (1)

o Female  (2)

Q34 How old are you?

o 18-30  (1)

o 30-50  (2)

o 50-65  (3)

o 65 or older  (4)

Q18 Please provide your email address.  We will use your email to let you know whether you are eligible for this study.  We will not use it for any other purpose.

Q15 Please re-enter your email address.

End of Block: Demographics

Start of Block: Debriefing

Q41 Thank you so much for filling out this questionnaire!  We will be in touch to let you know whether you are eligible for this study.

If you have any questions, you can contact Julia, the researcher in charge of this study, at ppolharvard@gmail.com.

End of Block: Debriefing

**Baseline**

Congratulations!  Based on your responses to the web survey that you completed, you are invited to participate in a study of how sports fans make decisions.  If you would like to learn more about participating in this study, please type your unique invitation code and proceed to the next page:

________________________________________________________________

Thank you for choosing to participate in this study!

In the rest of this short survey, you will:

(1) Provide your phone number, so that we can send you a text message after upcoming NFL games.

(2) Answer a few questions about yourself.

(3) Complete a series of short "balloon" tasks.  Each of these tasks will involve an opportunity to earn real money.  These tasks will be similar to the short surveys that you will complete after the upcoming football games.

In order to participate in this study, you must have a cell phone that can receive text messages.  We will use your cell phone number to send you text messages after upcoming football games to remind you that you will have 20 minutes to  complete the survey.  We will keep your information strictly  confidential, and we will not use your number for any other purpose.

What is your cell phone number? : ___________________________________________

In order to participate in this study, you must have, or obtain, a PayPal account. We will make payments to you through your Paypal account.

If you have a Paypal account, please enter your Paypal account email address here, otherwise type "I WILL GET AN ACCOUNT SHORTLY." If you don't have an account yet, please sign up and we will follow up with you to get your Paypal information in the next few days.

________________________________________________________________

What is your combined annual household income?

o Less than $40,000  (1)

o $40,000 – $59,999  (2)

o $60,000 – $89,999  (3)

o More than $90,000  (4)

What is the highest level of education you have completed?

o Did not complete high school  (1)

o High school / GED  (2)

o Some college  (3)

o 2-year or 4-year college degree  (4)

o Graduate degree  (5)

Do you play fantasy football?

o Yes, I am a passionate fantasy football player  (1)

o Yes, but I'm not too dedicated to it  (2)

o No  (3)

Is one or more of your fantasy teams involved in an important match-up this week?

o Yes, I'm in the fantasy football playoffs in one or more of my leagues  (1)

o Yes, but it’s not in the playoffs  (2)

o No  (3)

o I don't play fantasy football  (4)

If you do play fantasy football, how many leagues are you in? (please enter a number)

________________________________________________________________

BART Intro

In the rest of this survey, you will play three short "balloon" games.  These are very similar to the survey tasks that you will be asked to play after each NFL game.

In each balloon game, you will decide how far to inflate a balloon.  The more air you add, the more you will get paid if the balloon doesn't burst.  However, if the balloon pops, you will lose all of your money from that game.  You will begin each game with $1 for free.

Each game is for real money.  You will be paid online to your PayPal account.

BART Choice

Game #${e://Field/game}, Round #${e://Field/winnings}

If you stop now you will earn $(Winnings)}.

If you add air to the balloon, you could earn an additional $1.  However, if you add air, there is a (Probability)% chance that the balloon will pop.  If it pops, you will not earn any money this game.

 Do you want to add air to the balloon?

o Yes, try to add air!  (1)

o No, I will keep the money I've earned this game  (2)

BART Loss

Unfortunately, the balloon popped!  You will not earn any money this game.

Click next to proceed.

BART Keep

You chose to stop inflating the balloon.  Your earnings from this game $(Winnings) will be added to your payment.

Click next to proceed.

Bart Win

Congratulations!  You have inflated the balloon all the way without it popping.  Your earnings from this game $(Winnings) will be added to your payment.

Click next to proceed.

(For brevity, BART Choice, Loss, Keep and Win will be condensed into BART TASK going forward)

Emotions

Please describe your feelings right now.

|  | Does not describe my feelings (1) | (2) | (3) | Somewhat describes my feelings (4) | (5) | (6) | Describes my feelings (7) |
| --- | --- | --- | --- | --- | --- | --- | --- |
| Upset (1) | o | o | o | o | o | o | o |
| Excited (2) | o | o | o | o | o | o | o |
| Happy (3) | o | o | o | o | o | o | o |
| Irritable (4) | o | o | o | o | o | o | o |
| Sad (5) | o | o | o | o | o | o | o |
| Nervous (6) | o | o | o | o | o | o | o |
| Angry (7) | o | o | o | o | o | o | o |
| Disappointed (8) | o | o | o | o | o | o | o |

This coming weekend, your favorite NFL team (as reported on the previous survey) is playing.  What do you think is the chance that they will win?

o Likely to win.  (1)

o Could go either way.  (2)

o Likely to lose.  (3)

 Please predict the final score for this weekend's game, between your favorite team and their opponent, and write it down in this format - "YOURTEAM SCORE, OPPONENT SCORE"

________________________________________________________________

 Thank You

Thank you again for agreeing to participate in this research study, which will involve completing short surveys after a few upcoming NFL games.

We will contact you via email the day of the next football game to remind you to take the survey after the game.  We will also send you a text message after the game to let you know that you will have 20 minutes to complete the survey.

If you have questions about your participation, you can contact the researcher  in charge of this study, Syon Bhanot (syon_bhanot@hksphd.harvard.edu).  You can  also contact the Harvard faculty member supervising this work, Francesca  Gino (fgino@hbs.edu).

Your earnings from today's survey $(Winnings) from Balloon Game #1,  $(Winnings) from Balloon Game #2, and $(Winnings) from  Balloon Game #3) will be added to your electronic gift card, and will be  available in three days.

**S1.C. Post-Game Survey.** Post-game survey captures risk preferences and emotional state after game outcomes.

Thank you for participating in this research study of sports fans!  This survey will take approximately five minutes to complete.  You will be paid $5 for completing the survey, and you will also have the chance to earn up to **$26** in additional money.  To participate, please click next.

BART Intro

In the rest of this survey, you will play three short games.  In each game, you will decide how far to inflate a balloon.  The more air you add, the more you will get paid if the balloon doesn't burst.  However, if the balloon pops, you will lose all of your money from that game.  You will begin each game with $1 for free.

(BART TASK)

Emotions

|  |
| --- |

Please recall the football game that you watched today, and describe how you felt immediately after the game ended.

|  | Does not describe my feelings (1) | (2) | (3) | Somewhat describes my feelings (4) | (5) | (6) | Describes my feelings (7) |
| --- | --- | --- | --- | --- | --- | --- | --- |
| Excited (1) | o | o | o | o | o | o | o |
| Happy (2) | o | o | o | o | o | o | o |
| Proud (3) | o | o | o | o | o | o | o |
| Active (4) | o | o | o | o | o | o | o |
| Alert (5) | o | o | o | o | o | o | o |
| Strong (6) | o | o | o | o | o | o | o |
| Attentive (7) | o | o | o | o | o | o | o |
| Inspired (8) | o | o | o | o | o | o | o |
| Scared (9) | o | o | o | o | o | o | o |
| Sad (10) | o | o | o | o | o | o | o |
| Nervous (11) | o | o | o | o | o | o | o |
| Angry (12) | o | o | o | o | o | o | o |
| Disappointed (13) | o | o | o | o | o | o | o |

Please recall the football game that you watched today, and describe what you thought immediately after the game ended.

|  | None (1) | (2) | Some (3) | (4) | Quite a lot (5) | (6) | An Extreme Amount (7) |
| --- | --- | --- | --- | --- | --- | --- | --- |
| How certain were you about the consequences of the game? (1) | o | o | o | o | o | o | o |
| How pleasant was it for you to be engaged with this game as a fan? (2) | o | o | o | o | o | o | o |
| To what extent did you feel that the events were beyond anyone’s control? (3) | o | o | o | o | o | o | o |
| To what extent did you find the outcome "unexpected"? (4) | o | o | o | o | o | o | o |
| To what extent did you want the game to result in a pleasurable outcome for you (e.g. your team winning)?” (5) | o | o | o | o | o | o | o |
| To what extent did you want the game to just not result in a painful outcome to you (e.g. your team losing)? (6) | o | o | o | o | o | o | o |

Around how much of your favorite NFL team's game did you watch today?

o None, basically  (1)

o Less than half  (2)

o About half  (3)

o Most of it  (4)

o Every play  (5)

If you did NOT see every play of your favorite NFL team's game, why was that?

o Was busy with other activities and responsibilities  (1)

o I was flipping between games, or watching NFL Red Zone (or another premium channel that cuts between games)  (2)

o The game was boring  (3)

o I got frustrated watching the game as a fan  (4)

o Other  (5)

What do you think is the chance that your favorite NFL team will win next week's matchup?

o Likely to win.  (1)

o Could go either way.  (2)

o Likely to lose.  (3)

Please make a prediction of how your favorite NFL team will do next week, and write down the score using the following format (e.g. "Bengals 24, Giants 21):

________________________________________________________________

Do you play fantasy football?

o Yes, passionately  (1)

o Yes, but I'm not too into it  (2)

o No  (3)

Did any of your fantasy teams have an important match-up this week?

o Yes, I was in the fantasy playoffs in one or more leagues  (1)

o Yes, but it was not in the fantasy playoffs  (2)

o No  (3)

Please let us know how your fantasy team/teams did this week using text - the more detail the better  (e.g. "I won my championship in one league by a large margin this week and lost a close match-up in the 3rd/4th match-up in another league" etc.):

________________________________________________________________

Thank You

 Thank you for completing this survey!  Your participation payment of $5 and your additional earnings of $(Winnings) from Game #1, $(Winnings) from Game #2, and $(Winnings) from Game #3 will be added to your electronic gift card, and will be available in three days.  We will contact you again next week to remind you to complete the survey after the next big game!  Please contact us if you have any questions about your participation.

**S1.D Regular Season Surveys**

Disappointment_Online_Main_Study_After_Games_1

Disappointment_Online_Main_Study_After_Game_2_Week_17

I-1 Thank you for participating in this research study of sports fans!  This survey will take approximately five minutes to complete.  You will be paid $5 for completing the survey, and you will also have the chance to earn up to **$24** in additional money.  Please type your unique **participant ID number** and proceed to the next page to participate.

________________________________________________________________

BART Intro

I-2 In this survey, you will play three short games.  In each game, you will decide how far to inflate a balloon.  The more air you add, the more you will get paid if the balloon doesn't burst.  However, if the balloon pops, you will lose all of your money from that game.  You will begin each game with $1 for free.

(BART TASK)

Emotions

Q25 Which NFL team were you rooting for in the game you just watched (your favorite team as stated in the earlier surveys)?

(List NFL teams)

E How did you feel immediately after the (TEAM) game ended?  (0=didn't feel this emotion; 100=strongly felt this emotion)

|  | 0 | 100 |
| --- | --- | --- |

| Excited (1) | Slider/100 |
| --- | --- |
| Happy (2) | Slider/100 |
| Proud (3) | Slider/100 |
| Sad (10) | Slider/100 |
| Nervous (11) | Slider/100 |
| Angry (12) | Slider/100 |
| Disappointed (13) | Slider/100 |

Q43 Around how much of the (TEAM) game did you watch today?

o Almost none  (1)

o Less than half  (2)

o About half  (3)

o Most of it  (4)

o Every play  (5)

Q45 If the (TEAM) are playing next week, how likely are they to win?

o Likely to win  (1)

o Could go either way  (2)

o Likely to lose  (3)

 Q17 Do you play fantasy football?

o Yes  (1)

o No  (2)

Display Q18: If Do you play fantasy football? = Yes

Q18 Did any of your fantasy teams have an important match-up this week?

o Yes, I was in the fantasy playoffs in one or more leagues  (1)

o Yes, but it was not in the fantasy playoffs  (2)

o No  (3)

Q15 Please enter your CONTACT email address, as you did in the previous surveys.

________________________________________________________________

Thank You

Q41  Thank you for completing this survey!  Your participation payment of $5 and your additional earnings of $(Winnings)from Game #1, $(Winnings) from Game #2, and $(Winnings) from Game #3 will be added to your PayPal account within the next 48 hours.  We will contact you again next week to remind you to complete the survey after the next big game!  Please contact us if you have any questions about your participation.

**Disappointment_Online_Main_Study_After_Games_3_**

I-1 Thank you for participating in this research study of sports fans!  This survey will take approximately five minutes to complete.  You will be paid $5 for completing the survey, and you will also have the chance to earn up to **$34** in additional money.  Please type your unique participant ID number and proceed to the next page to participate.

________________________________________________________________

BART Intro

I-2 In this survey, you will play three short games.  In each game, you will decide how far to inflate a balloon.  The more air you add, the more you will get paid if the balloon doesn't burst.  However, if the balloon pops, you will lose all of your money from that game.  You will begin each game with $2 for free.

Q14 Please read each question carefully!  The probability that the balloon pops changes across games.  The amount of money that you earn from successfully adding air also changes across games.

(BART TASK)

Emotions

Q25 Which NFL team were you rooting for in the game you just watched (your favorite team as stated in the earlier surveys)?

(List NFL teams)

E How do you feel right now?  (0=don't feel this emotion; 100=strongly feel this emotion)

|  | 0 | 100 |
| --- | --- | --- |

| Excited (1) | Slider/100 |
| --- | --- |
| Happy (2) | Slider/100 |
| Proud (3) | Slider/100 |
| Sad (10) | Slider/100 |
| Nervous (11) | Slider/100 |
| Angry (12) | Slider/100 |
| Disappointed (13) | Slider/100 |

Q43 Around how much of the (TEAM) game did you watch today?

o Almost none  (1)

o Less than half  (2)

o About half  (3)

o Most of it  (4)

o Every play  (5)

Q15 Please enter your CONTACT email address, as you did in the previous surveys.

________________________________________________________________

Thank You

Q41  Thank you for completing this survey!  Your participation payment of $5 and your additional earnings of $(Winnings)from Game #1, $(Winnings) from Game #2, and $(Winnings) from Game #3 will be added to your PayPal account within the next 48 hours.  We will contact you again next week to remind you to complete the survey after the next big game!  Please contact us if you have any questions about your participation.

**S1.E Playoff Weekend Surveys**

I-1 Thank you for participating in this research study of sports fans!  This survey will take approximately five minutes to complete.  You will be paid $5 for completing the survey, and you will also have the chance to earn up to **$34** in additional money.  Please type your unique **participant ID number** and proceed to the next page to participate.

________________________________________________________________

BART Intro

I-2 In this survey, you will play three short games.  In each game, you will decide how far to inflate a balloon.  The more air you add, the more you will get paid if the balloon doesn't burst.  However, if the balloon pops, you will lose all of your money from that game.  You will begin each game with $2 for free.

Q14 Please read each question carefully!  The probability that the balloon pops changes across games.  The amount of money that you earn from successfully adding air also changes across games.

(BART TASK)

Emotions

Q25 Which NFL team were you rooting for in the game you just watched (your favorite team as stated in the earlier surveys)?

(List NFL teams)

E How do you feel right now?  (0=don't feel this emotion; 100=strongly feel this emotion)

|  | 0 | 100 |
| --- | --- | --- |

| Excited (1) | Slider/100 |
| --- | --- |
| Happy (2) | Slider/100 |
| Proud (3) | Slider/100 |
| Sad (10) | Slider/100 |
| Nervous (11) | Slider/100 |
| Angry (12) | Slider/100 |
| Disappointed (13) | Slider/100 |

Q43 Around how much of the (TEAM) game did you watch today?

o Almost none  (1)

o Less than half  (2)

o About half  (3)

o Most of it  (4)

o Every play  (5)

Q15 Please enter your CONTACT email address, as you did in the previous surveys.

________________________________________________________________

Thank You

Q41  Thank you for completing this survey!  Your participation payment of $5 and your additional earnings of $(Winnings) from Game #1, $(Winnings) from Game #2, and $(Winnings) from Game #3 will be added to your PayPal account within the next 48 hours.  We will contact you again next week to remind you to complete the survey after the next big game!  Please contact us if you have any questions about your participation.
